# Mitochondrial genomes and comparative analyses of *Culex camposi, Culex coronator, Culex usquatus* and *Culex usquatissimus* (Diptera:Culicidae), members of the coronator group

**DOI:** 10.1186/s12864-015-1951-0

**Published:** 2015-10-21

**Authors:** Bruna Demari-Silva, Peter G. Foster, Tatiane M. P. de Oliveira, Eduardo S. Bergo, Sabri S. Sanabani, Rodrigo Pessôa, Maria Anice M. Sallum

**Affiliations:** Departamento de Epidemiologia, Faculdade de Saúde Pública, Universidade de São Paulo, São Paulo, Brazil; Department of Life Sciences, Natural History Museum, Cromwell Road, London, England; Superintendência de Controle de Endemias, Secretaria de Estado da Saúde de São Paulo, Araraquara, São Paulo Brazil; Department of Pathology, LIM 03, Hospital das Clínicas (HC), School of Medicine, University of São Paulo, São Paulo, Brazil

**Keywords:** *Culex*, Coronator Group, Mitochondrial genome, Arboviruses, Vectors

## Abstract

**Background:**

The Coronator Group currently encompasses six morphologically similar species (*Culex camposi* Dyar, *Culex coronator* Dyar and Knab, *Culex covagarciai* Forattini, *Culex usquatus* Dyar, *Culex usquatissimus* Dyar, and *Culex ousqua* Dyar). *Culex coronator* has been incriminated as a potential vector of West Nile Virus (WNV), Saint Louis Encephalitis Virus (SLEV), and Venezuelan Equine Encephalitis Virus (VEEV). The complete mitochondrial genome of *Cx. coronator*, *Cx. usquatus, Cx.usquatissimus,* and *Cx. camposi* was sequenced, annotated, and analyzed to provide genetic information about these species.

**Results:**

The mitochondrial genomes of *Cx. coronator*, *Cx. usquatus, Cx.usquatissimus,* and *Cx. camposi* varied from 15,573 base pairs in *Cx. usquatus* to 15,576 in *Cx. coronator*. They contained 37 genes (13 protein-encoding genes, 2 rRNA genes, and 22 tRNA genes) and the AT-rich control region. Comparative analyses of the 37 genes demonstrated the mitochondrial genomes to be composed of variable and conserved genes. Despite the small size, the *ATP8*, *ATP6* plus *NADH5* protein-encoding genes were polymorphic, whereas tRNAs and rRNAs were conserved. The control region contained some poly-T stretch. The Bayesian phylogenetic tree corroborated that both the Coronator Group and the *Culex pipens* complex are monophyletic taxa.

**Conclusions:**

The mitochondrial genomes of *Cx. coronator*, *Cx. usquatus, Cx. usquatissimus* and *Cx. camposi* share the same gene composition and arrangement features that match to those reported for most Culicidae species. They are composed of the same 37 genes and the AT-rich control region, which contains poly-T stretches that may be involved in the functional role of the mitochondrial genome. Taken together, results of the dN/dS ratios, the sliding window analyses and the Bayesian phylogenetic analyses suggest that *ATP6, ATP8* and *NADH5* are promising genes to be employed in phylogenetic studies involving species of the Coronator Group, and probably other species groups of the subgenus *Culex*. Bayesian topology corroborated the morphological hypothesis of the Coronator Group as monophyletic lineage within the subgenus *Culex*.

**Electronic supplementary material:**

The online version of this article (doi:10.1186/s12864-015-1951-0) contains supplementary material, which is available to authorized users.

## Background

*Culex camposi* Dyar, *Culex coronator* Dyar and Knab, *Culex covagarciai* Forattini, *Culex ousqua* Dyar, *Culex usquatus* Dyar and *Culex usquatissimus* Dyar are members of the Coronator Group of the subgenus *Culex* [1]. Morphological features of the fourth-instar larva, pupa and female cannot distinguish among species of the Coronator Group. However, traits of the male genitalia allow species identification. Species of the Coronator Group are largely distributed and some are sympatric in the Neotropics [2]. In southeastern Brazil, for instance, the geographical distribution of *Cx. usquatus, Cx. coronator* and *Cx. camposi* overlaps [1]*.* Phylogenetic relationships among the species remain unresolved, because results of studies employing DNA sequences of the cytochrome c oxidase subunit 1 (*COX1*) mitochondrial gene [3, 4] and of the internal transcribed spacer 2 (ITS2) of the ribosomal DNA [5] showed poorly supported clades. Likely because of the lack of studies, there is no evidence of hybridization among species of the Coronator Group.

*Culex coronator* is a potential vector of arboviruses. As such, this species may participate in the dynamics of the transmission of the Saint Louis Encephalitis Virus (VESL), the Venezuelan Equine Encephalitis Virus (VEEV), the Mucambo Virus (MV) [6, 7], and the West Nile Virus (WNV) [8]. Experiments of susceptibility of *Cx. coronator* to the WNV showed that the population from Florida, USA, is competent in disseminating the WN-FL03p2-3 strain under some conditions; however, the transmission rate decreases at lower temperatures (0–17 % at 25 °C and 28–67 % at 28 °C) [8]. The impossibility of accurately identifying a species can lead to inconclusive studies regarding the vector status of it. The problematic identification of species of the Coronator Group may be causing difficulties in defining the medical importance of this group.

The mitochondrial genome of the majority of the metazoan organisms is a small genome (ranging from 15 to 20 kb). It contains 37 genes, which encode 13 Protein-Coding Genes (PCGs) involved in oxidative phosphorylation, 2rRNA (*rrnL* and *rrnS*) and 22 tRNAs genes, which are necessary for the translation of the proteins encoded by the genes [9, 10]. The mitochondrial genome also encompasses a region that is rich in adenine (A) and thymine (T). This AT-rich region starts and controls the replication of the mitochondrial genome [11]. Because of the lack of recombination and fast evolutionary processes in comparison to those that involve nuclear genes, the mitochondrial genome seems to be a source of information that can be employed to address evolutionary processes of both vertebrates and invertebrates [12–14].

Although being largely employed in molecular taxonomy and phylogeny, only *Culex quinquefasciatus* Say and *Culex pipiens* L. possess their mitochondrial genome available in the Genbank. Considering the potential taxonomic and phylogenetic content of genes of the mitochondrial genome for *Culex* species and the role of *Cx. coronator* as vector of arboviruses, four species of the Coronator Group were the focus of this study. Next generation sequencing technology was applied to sequence the mitochondrial genomes of *Culex coronator*, *Culex usquatus*, *Culex camposi*, and *Culex usquatissimus*. The objectives of the study were to: 1) describe the mitochondrial genome of four species of the Coronator Group; 2) to analyze the mitochondrial genome of four species of the Coronator Group; 3) to compare the genomes of species of the Coronator Group with other species of the Culicidae family available in the Genbank; 4) to assess the monophyly of the Coronator Group.

## Methods

### Mosquito sampling and DNA extraction

**Fig. 1:**
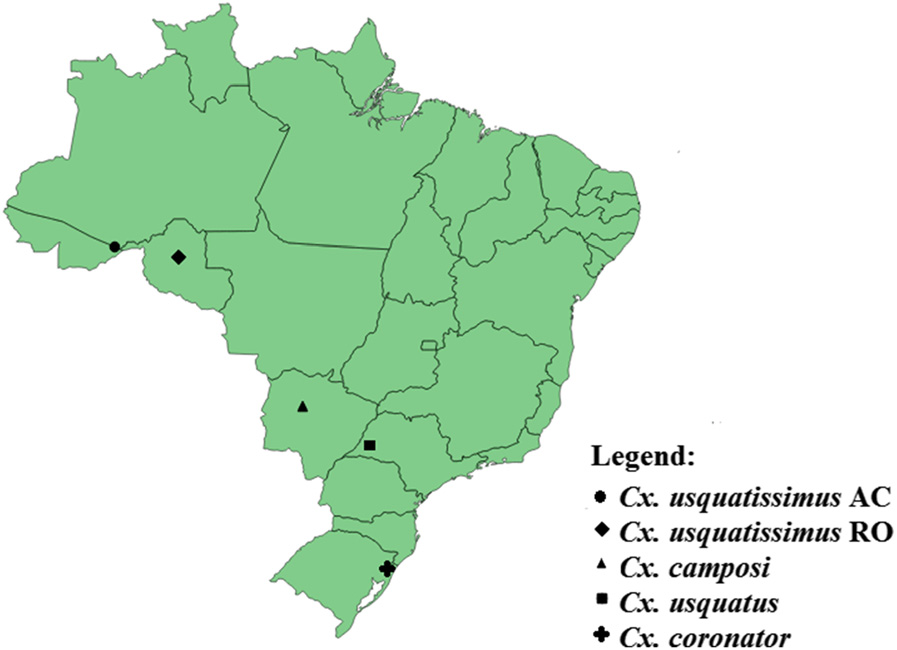
Map of Brazil showing the collection sites of species of the Coronator Group of *Culex* (*Culex*) (Diptera: Culicidae)

Mosquitoes and collection data from individuals employed in the study are in Table 1 and Fig. 1. Immature stages were field collected as either larvae or pupae and kept in the laboratory to obtain adults associated with larval and pupal exuviae for species identification. Only males were employed to sequence the mitochondrial genome because species identification is based on traits of the male genitalia (Table 1). Fourth-instar larval, pupal exuviae and dissected male genitalia were mounted on microscope slides in Canada balsam and are deposited in Coleção Entomológica de Referência, Faculdade de Saúde Pública, Universidade de São Paulo, Brazil, as vouchers. Males were kept in 95 % ethanol and stored at −80 °C until DNA extraction.

**Table 1:** Species, specimen codes, gender, states and geographical coordinates of the collection of localities in Brazil

Species	Specimencode	Sex	State	Geographicalcoordinates
*Culex coronator*	RS10_109	♂	Rio Grande do Sul	29°39′35″S 50°13′03″W
*Culex usquatissimus*AC	AC16_101	♂	Acre	9°41′03″S 67°08′05,3″W
*Culex usquatissimus*RO	RO25_19	♂	Rôndonia	10°18″03″S 63°14′09,1″W
*Culex usquatus*	SP29_156	♂	São Paulo	21°37′07″S 50°56′24″W
*Culex camposi*	MS04_38	♂	Mato Grosso do Sul	19°29′59,4″S 55°36′33,8″W

### PCR amplification and sequencing

Genomic DNA was extracted from each mosquito individually using QIAgen Dneasy® Blood and Tissue kit (QIAgen Ltd., Crawley, UK) following the same procedures adopted by Foster et al. [15]. The mitochondrial genome of each individual was amplified in two PCRs. A long PCR amplification generated a fragment of ~12,000 base pairs (bp) and a second reaction targeted a fragment of ~4,500 bp. Two sets of primers were used as follows: 16Sa (5′ CGCCTGTTTATCAAAAACAT 3′) and LCO1490 (5′ GGTCAACAAATCATAAAGATATTGG 3′) to amplify ~12,000-bp fragment, and 16Sb (5′ CCGGTTGAACTCAGATCA 3′) and HCO2198 (5′ TAAACTTCAGGGTGACCAAAAAATCA 3′) to generate fragments of ~4,500-bp. See Folmer et al. 1994 [16] for additional information about the LCO1490 and HCO 2198 primers, and Kambhampati and Smith (1995) [17] for 16Sa and 16Sb primers. Each PCR reaction was performed using GoTaq® Long PCR Master Mix (Promega, Wisconsin, USA) following the protocols proposed by the manufacturer. The long PCR products were purified in DNA Clean & Concentrator™ (Zymo Research, California, USA) and quantified with Qubit (LifeTechnologies, Oregon, USA) for library preparation. Individually barcoded PCR libraries were created for the 12,000 bp and 4,500 bp PCR products employing Nextera® XT DNA Sample Preparation Kit (Illumina, Illinois, USA) and sequenced on the Illumina MiSeq platform with paired-end 250 base pairs chemistry.

### Genome assembly and annotation

Mira v4 software was used for sequence assembly and Tablet to view the assembly [18, 19]. The MITOS website [20] was used to delimit the genes. Annotation of protein coding genes was conducted using the Wise2 v2.2 package and the HMMER2 models [21] (HMMER v3.1b2; http://hmmer.janelia.org/).

### Sequence analysis

Nucleotide composition values were calculated using the MEGA 6.06 software [22]. The GC—and AT-skews were used to determine strand asymmetry [23]. To measure these values, the following formulas were used: GC-skew (G-C) / (G + C) and AT-skew (A-T) / (A + T). The secondary structure of tRNAs was inferred in tRNAscan-SE 1.21 [24]. A sliding window of 200 bp and steps of 25 bp was implemented to estimate nucleotide divergence. The analysis was performed using the five complete mitochondrial genome sequences in DnaSP v. 5.10.01 [25]. Ratios between non-synonymous (dN) and synonymous (dS) substitution among protein-coding sequences of all pairs of species were calculated in KaKs calculator [26] using a modified Yang-Nielsen algorithm. This ratio is currently employed to estimate if genes are under negative (purifying) selection (dN/dS < 1), positive (adaptative) selection (dN/dS > 1), or neutral evolution.

### Phylogenetic analysis

Phylogenetic analysis was carried out using Bayesian inference from the concatenated sequences of all protein coding genes of the five newly sequenced *Culex* specimens, plus the mitochondrial genomes of four species available in the GenBank, as follows: *Cx. quinquefasciatus* [HQ724617], *Cx. pipiens* [HQ724616, HQ724615], *Aedes albopicutus* [NC_006817] and *Anopheles darlingi* [NC_014275]. The latter two species were employed as outgroups. Multiple alignments were generated in Clustal X [27] and then edited visually in BioEdit [28] to ensure that genes were in the frame. The best-fit model of nucleotide substitution for each gene was estimated in JModeltest 0.1.1 package [29], under Akaike Information Criterion (AIC). Bayesian analysis was performed in MrBayes v. 3.1.2 [30] and the dataset was partitioned by genes. Two independent runs with one cold and three heated chains each, were implemented for 1,000,000 generations with trees sampled every 100 generations; the consensus topology was generated after a burnin of 25 % of the retained trees.

## Results and discussion

### Mitochondrial genome organization and nucleotide composition

The mitochondrial genomes of *Cx. coronator, Cx. usquatus, Cx. usquatissimus* and *Cx. camposi* contain 37 genes, which have been found in other metazoan and Culicidae species, including 13 protein-encoding genes, 22 tRNAs and 2 rRNA genes ,( *rrnL* and *rrnS*) and the AT-rich control region [31–34]. The genomes are approximately of the same length of *Culex quinquefasciatus* (15,587 base pairs): 15,573 bp in *Cx. usquatus* and *Cx. usquatissimus* from Acre state (*Cx. usquatissimus* AC)*;* 15,574 bp in *Cx. usquatissimus* from Rondônia state (*Cx. usquatissimus* RO); and 15,576 bp in *Cx. coronator* (Table 2). No difference was observed in the length of the 13 protein coding genes. Furthermore, differences in gene length were primarily caused by insertion/deletions in the intergenic spacers and in the AT-control region.

**Table 2:** Structural feature of the mitochondrial genome of four species of *Cx. coronator, Cx. usquatus, Cx. camposi,* and *Cx. usquatissimus*

Species	Mitochondrial gene structure in base pairs				
	PCG	tRNA genes	rRNA genes	Control region	Total
*Cx. coronator*	11,226	1,482	2,124	725	15,576
*Cx. usquatissimus* AC	11,226	1,482	2,124	721	15,573
*Cx. usquatissimus* RO	11,226	1,483	2,124	722	15,574
*Cx. usquatus*	11,226	1,483	2,124	719	15,573
*Cx. camposi*	11,226	1,482	2,124	719	15,570

In all species, nucleotide composition was biased toward overall AT content. This AT-bias has been reported in the mitochondrial genomes of other metazoan species [34–37]. In *Cx. coronator, Cx. usquatissimus* AC from Acre state and *Cx. usquatissimus* RO from Rondônia state, the AT content value was 78.6 %*,* whereas in *Cx. usquatus* and *Cx. camposi* it was 78.9 % (Table 3). These values were similar to those found in other Culicidae species with the mitochondrial genome reported [31–34]. Considering species of the *Anopheles albitaris* complex, the AT content ranged from 77.1 % to 77.4 %; whereas in *Cx. quinquefasciatus* it was 77.7 %, and in *Ae. aegypti,* 79 %. Similar to *Anopheles* species [34] and other orders of Insecta [36], the value of the AT content increased in the third codon position of the PCGs varying from 81.4 % to 86.6 %, being slightly lower than in the control region (89.2 % to 90.1 %). One explanation for this phenomenon is the “transcription hypothesis of the codon usage” [38]. According to Sun et al. [38], the high availability of ATPs, along with the lack of other NTPs in cells, leads to the maximization of the use of adenines in the third codon position in order to increase the efficacy of the transcription. The overall AT-skew was positive in all species (0.0025 for *Cx. usquatissimus* RO and *Cx. usquatissimus* AC; 0.0012, 0.003 and 0.0038*,* in *Cx coronator, Cx. camposi* and *Cx. usquatus,* respectively), whereas the GC-skew was −0.15 for all species, showing that adenine and cytosine are in the highest proportion compared to thymine and guanine, respectively, in the majority strand. Those findings are in agreement with other studies of species of the genera *Anopheles* and *Drosophila* [34].

**Table 3:** Overall nucleotide composition and A + T content per structural feature, and by codon position in protein-coding genes

	%				%A + T					%A + T in PCG		
										Codon position		
	T	C	A	G	Overall	tRNA	rRNA	Control region	Intergenic space	1st	2nd	3nd
*Cx. usquatissimus* AC	39.2	12.4	39.4	9.1	78.6	78.9	82.1	90.1	94.5	78.0	67.2	86.3
*Cx. coronator*	39.2	12.4	39.3	9.1	78.6	79.1	82.2	89.4	89.5	78.3	67.2	86.6
*Cx. usquatissimus* RO	39.2	12.4	39.4	9.1	78.6	79.1	82.1	89.5	89.5	77.0	73.5	81.4
*Cx. usquatus*	39.2	12.4	39.5	9.0	78.7	78.6	82.1	89.2	90.9	78.3	67.2	86.4
*Cx. camposi*	39.2	12.3	39.5	9.0	78.7	79.1	82.4	89.6	89.6	77.1	73.5	81.5

### The protein-coding genes (PCGs)

Except for the *COX1* gene in which no canonical ATN was found, the majority of the PCGs of *Culex* species possessed the start codon ATN (Additional file 1). The absence of the ATN start codon in the *COX1* gene was found in *Cx. quinquefasciatus, Cx. pipiens, Ae. aegytpti* and in other insects [31–35] and therefore it has been widely discussed [39]. In 1983, de Brujin [40] proposed a tetranucleotide (ATAA) positioned upstream in the proximity of the opening codon of *COX1* (TCG) as a start codon for *Drosophila melanogaster.* The author hypothesized that if an ATA, a recognized start codon, overlaps a stop codon TAA in the frame −1, the ATA of the tetranucleotide ATAA could function as a highly conserved 0 frame and as an opening frame. Notwithstanding, in the mitochondrial genome of *Culex* species, the closest ATAA tetranucleotide is located within the t*rnL2* gene, overlapping 36 base pairs of the gene. Consequently, it is plausible to suppose that the hexanucleotide ATTTAA, which usually flanks the beginning of the *COX1* in mosquitoes, is involved in the initiation of the translation signal of this gene, as it was proposed for *Tetrodontophora bielanensis* [39].

Eleven genes (*ATP6*, *ATP8*, *COX3*, *CytB*, *NAD2*, *NAD3*, *NAD4*, *NAD4L*, *NAD5*, *NAD6*) have complete TAA stop codons, except for *COX1* and *COX2* genes, which terminate with an incomplete codon T. This phenomenon has been extensively reported in other mitochondrial genomes, especially in *COX* genes, and so far it has been related to posttranscriptional polyadenylation, during which residual adenines are added in order to originate TAA terminators [34, 36, 41].

**Fig. 2:**
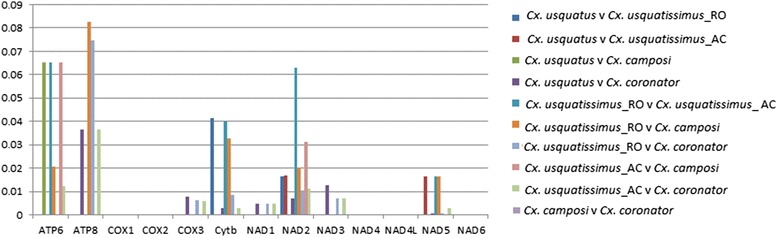
Ratio of non-synonymous / synonymous (dN/dS) nucleotide substitutions. Ratios were calculated among the 13 protein-coding genes of the mitochondrial genome of four species of the subgenus *Culex*: *Cx. coronator, Cx. usquatus, Cx. camposi,* and *Cx. usquatissimus*

A total of 322 nucleotide sites were variable across PCGs of all *Culex* species analyzed, in which 270 were at the 3rd, 51 at the 1st and 1 at the 2nd codon position, encompassing most of those transition mutations. As expected, genes with a larger length (*NAD2, COX1, NAD4, NAD5, CytB* and *NAD1*) concentrated the majority of the polymorphisms; however *COX1,*only presented synonymous substitutions, whereas *NAD2*, *NAD5*, *NAD4*, *CytB,* and *NAD1* showed 3, 2, 1, 3, and 2 non-synonymous substitutions, respectively. Additionally, despite the small length, *ATP6* and *ATP8* were polymorphic genes in *Culex* species, with 23 (2 non-synonymous) and 4 variable (2 non-synonymous) sites. Additionally, the dN/dS ratios (Fig. 2) were very low, ranging from 0.0 to 0.09, suggesting that all PCGs are under strong negative selection. Despite that, the strength of selection varies among genes; *COX1*, *COX2*, *COX3*, *NADH1*, *NADH3*, *NADH4*, *NADH4L* and *NADH6* are the mitochondrial genes that are under the strongest purifying selection.

Results of the analysis of the relative synonymous codon usage (RSCU) showed that the codons with adenine and thymine in the third position were overused when compared to other synonymous codons (Table 4), which commonly occurs in other metazoan mitochondrial genomes [36, 37, 42]. This can be observed in the leucine amino acid, i.e., while the TTA codon presented a RSCU value of 3.75, the codon CTG, which also translates a leucine, showed a RSCU value of 0.14.

**Table 4:** Relative synonymous codon usage (RSCU) and overall average of codon usage in the PCGs of *Cx. coronator, Cx. usquatus, Cx. camposi,* and *Cx. usquatissimus*

Amino acid	Codon	Overall average	RSCU	Amino acid	Codon	Overall average	RSCU	Amino acid	Codon	Overall average	RSCU	Amino acid	Codon	Overall average	RSCU
F	TTT	218.2	1.51	S	TCT	59.6	1.39	Y	TAT	156.4	1.5	C	TGT	36.0	1.2
F	TTC	71.4	0.49	S	TCC	38.4	0.89	Y	TAC	51.4	0.5	C	TGC	24.6	0.8
L	TTA	297.6	3.75	S	TCA	86.4	2.01	stop	TAA	-	-	W	TGA	75.2	1.6
L	TTG	33.4	0.42	P	CCT	9.6	0.22	stop	TAG	-	-	W	TGG	16.4	0.4
L	CAA	50.4	0.64	P	CCC	49.6	2.00	H	CAT	63.4	1.5	R	CGT	2.2	0.3
L	CTC	19.8	0.25	P	CCA	17.0	0.68	H	CAC	21.2	0.5	R	CGC	1.0	0.1
L	CTA	63.4	0.80	P	CCA	31.2	1.26	Q	CAA	68.0	1.7	R	CGA	26.4	3.5
L	CTG	11.0	0.14	P	CCG	1.6	0.06	Q	CAG	12.0	0.3	R	CGG	1.0	0.1
I	ATT	274.6	1.62	T	ACT	91.4	1.93	N	AAT	240.4	1.5	S	AGT	57.0	1.3
I	ATC	63.8	0.38	T	ACC	18.2	0.38	N	AAC	74.2	0.5	S	AGC	30.2	0.7
M	ATA	211.6	1.68	T	ACA	74.8	1.58	K	AAA	204.2	1.6	S	AGA	38.4	0.9
M	ATG	39.6	0.32	T	ACG	5.0	0.11	K	AAG	54.2	0.4	S	AGG	23.8	0.6
V	GTT	48.6	1.56	A	GCT	60.2	2.68	D	GAT	32.0	1.6	G	GGU	17.2	0.7
V	GTC	2.8	0.09	A	GCC	6.0	0.27	D	GAC	8.0	0.4	G	GGC	0.8	0.0
V	GTA	71.8	2.30	A	GCA	23.2	1.03	E	GAA	54.8	1.6	G	GGA	80.4	3.1
V	GTG	1.8	0.06	A	GCG	0.4	0.02	E	GAG	12.0	0.4	G	GGG	5.6	0.2

### RNA genes

The set of 22 tRNA genes in all species varied in length from 64 bp in *trnR* gene to 72 bp in *trnV* gene (Additional file 1)_._ The AT content ranged from 78.6 % to 79.1 % (Table 3). The anticodons of all tRNAs in *Culex* species were the same as that of other insects [31–35] (Additional file 1). The conventional cloverleaf-like structure of tRNAs was found in 20 (Additional file 2) of the 22 tRNAs described for *Culex* species. In contrast, the *trnR* and *trnS1* genes could not be folded into cloverleaf-like structure. In the *trnS1*, several similar cases were reported in previously sequenced metazoan mitochondrial genomes because of the replacement of the DHU (dihydrouridine) arm by a DHU-loop [43, 44]. The absence of a secondary structure in the *trnS1* has been reported for *Collembola* species [39].

Similar to the mitochondrial genome of other metazoan species [32–37], the *rrnL* is located between *trnL1* and *trnV* ribosomal genes, and the *rrnS* is situated between the *trnV* gene and the AT-rich region. In all species, the *rrnL* and *rrnS* are 2,124 base pairs long (Additional file 1). The AT content was 82 %, a value that is slightly higher than the overall content, however, similar to that observed in the 3rd codon position (Table 3). Both tRNAs and rRNAs genes are more conserved than the PCGs, with only 10 variable sites in tRNAs (throughout 1,462pb - 1,463 bp) and 11 substitutions among 2,124 bp of the two rRNAs genes. Evolutionary studies relative to the tRNA and rRNA in *Drosophila* and *Anopheles* showed signatures of negative selection, because non-pairing substitutions such as G-C to G-U can affect the secondary structure of these genes [41, 45].

### Intergenic spacer sequences

A total of 52 bp to 56 bp non-coding nucleotides were found in the mitochondrial sequences of the species included in this study (Table 2). The length of each spacer mostly varies from 1 to 5 bp, except for two spacers: one located between the *trnk* and the *tranD* genes (12 bp in *Cx. coronator*, and 11 bp in the remaining species); and the other between *trnS2* and *NAD1* genes with 19 bp in all samples. Although those spacers account for only 0.3 % of the whole mitochondrial genomes, they are composed almost exclusively of adenine and thymine, with an AT content that is higher than that observed in the AT-control region, reaching 94.5 % in *Cx. usquatissimus* from Acre (Table 3).

### AT-rich control region

The size variation, ranging from 719 bp in *Cx. usquatus* to 724 bp in *Cx. coronator* (Table 2) is within the range variation observed in the mitochondrial genomes reported for other Culicidae (from 531 bp in *Anopheles gambiae* to 731 bp in *Culex quinquefasciatus*). Those differences in length were caused by several indels of one to three nucleotides distributed throughout the AT regions of the mitochondrial genomes. Considering both indels and nucleotide substitutions, 34 variable sites were observed in *Culex* species. Similar to nuclear noncoding DNA, the AT-control region is subjected to a high level of polymorphisms that lead to variation in size and base composition. However, in species of the *Anopheles gambiae* complex, there is evidence that this region change more slowly than the 3rd codon position of PCGs [46]. Additionally, among different subgenera of *Anopheles,* there are conservative poly-T regions of 15 to 18 bp [34]. Comparing the control region of the four *Culex* species studied, we observed 4 conservative T-stretch regions of 6 to 15 bp. This was likely caused because this region contains several regulatory elements, including the origin of replication and transcription of the major strand of the mitochondrial genome [37] and, consequently, has a functional role, which is conserved among genera.

### Sliding window analysis

**Fig. 3:**
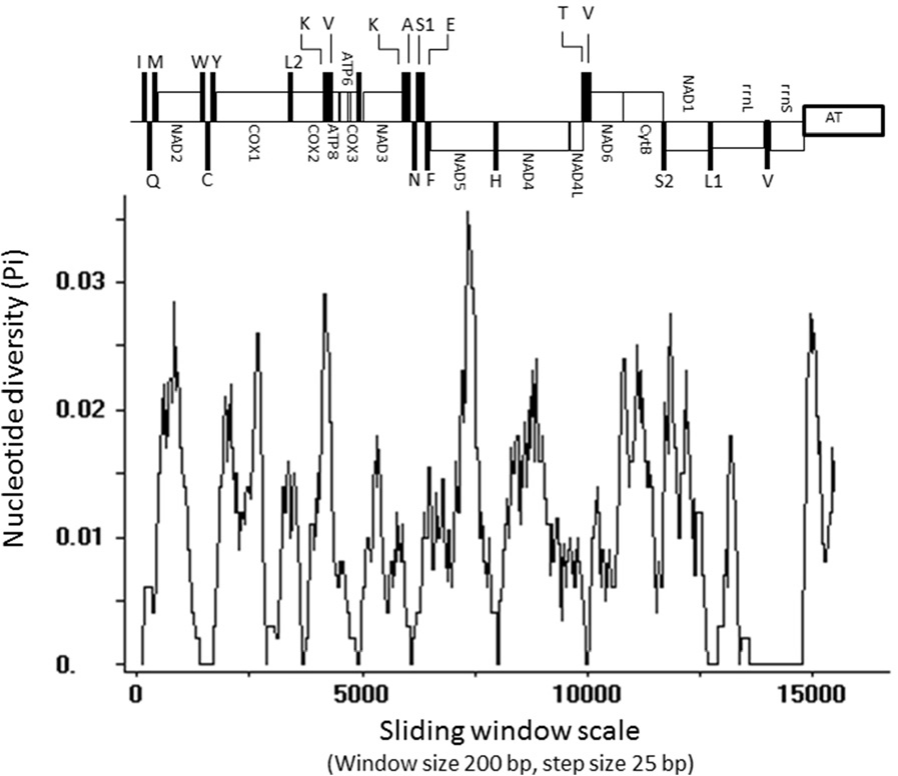
Graphics showing the nucleotide diversity of the mitochondrial genome of the four species. Nucleotide diversity of *Cx. coronator, Cx. usquatus, Cx. camposi* and *Cx. usquatissimus* was calculated using a sliding window analytical procedure (window of 200 base pairs with step size of 25 base pairs). Gene boundaries are indicated above the graph as follows: black vertical bars indicate tRNA genes, whereas the white boxes represent both protein-coding genes and ribosomal RNA genes. The featured rectangle shows the AT-rich control region

Mitochondrial DNA is largely employed in phylogeny and evolutionary studies because it has a high mutation rate, maternal inheritance, high number of copies, and intraspecific polymorphisms [47]. Herein, we conducted a sliding window analysis using the alignment of the entire mitochondrial genome of *Culex* species to verify regions with high nucleotide divergence and thus, identified potential markers that can be employed for future studies focusing on *Culex* species. Results of the sliding window analysis showed low nucleotide diversity (Pi), ranging from 0.0 to 0.035 (Fig. 3) among species of the Coronator Group. However, nucleotide substitution rates varied across both mitochondrial genomes and within individual genes. Remarkably, the 5′ end of the *NADH5* fragment showed the highest nucleotide diversity (0.035) among all mitochondrial genes. The *COX1* presented low variation, mainly in its 5′ proximal half. This region is largely employed in studies focusing in Culicidae species, and it is considered as the universal barcode for species identification [48]. Notwithstanding, other descriptive studies focusing on the mitochondrial genome showed that the *COX1* is a conserved gene [49–53]. Consequently its utility as a universal barcode needs to be reviewed.

The *rrnL* and *rrnS* demonstrated to be the most conserved genes within the mitochondrial genomes of species of the Coronator Group, showing low nucleotide diversity. Although *rrnL* usually shows high variability among some groups, i.e., Taeniidae [49], Nematoda [50], and Octopodus [54], herein, the highest nucleotide diversity found in the *rrnL* was 0.02*.* Per se, the *rrnL* and *rrnS* genes seem not to be good markers for taxonomy and evolutionary studies focusing on species of the Coronator Group.

Considering both the epidemiological importance of *Cx. coronator* and the problematic identification of females based on morphological characters, the development of molecular markers can facilitate species identification as well as establish phylogenetic relationships within the group and other closely related species. Taken together, results of the sliding window analysis and the dN/dS ratios indicate that the *NADH5, ATP8* and *ATP6* genes represent potential markers for future investigations regarding species of the Coronator Group, primarily because they retain high variations among PCGs with an elevated proportion of non-synonymous substitutions. Notwithstanding, the low levels of diversity shown herein, demonstrates that the utility of sequences of the mitochondrial gene needs further evaluation in future studies employing a larger sample size and other species of the subgenus *Culex* as well as other *Culex* (*Culex*) species.

### Phylogenetic analysis

**Fig. 4:**
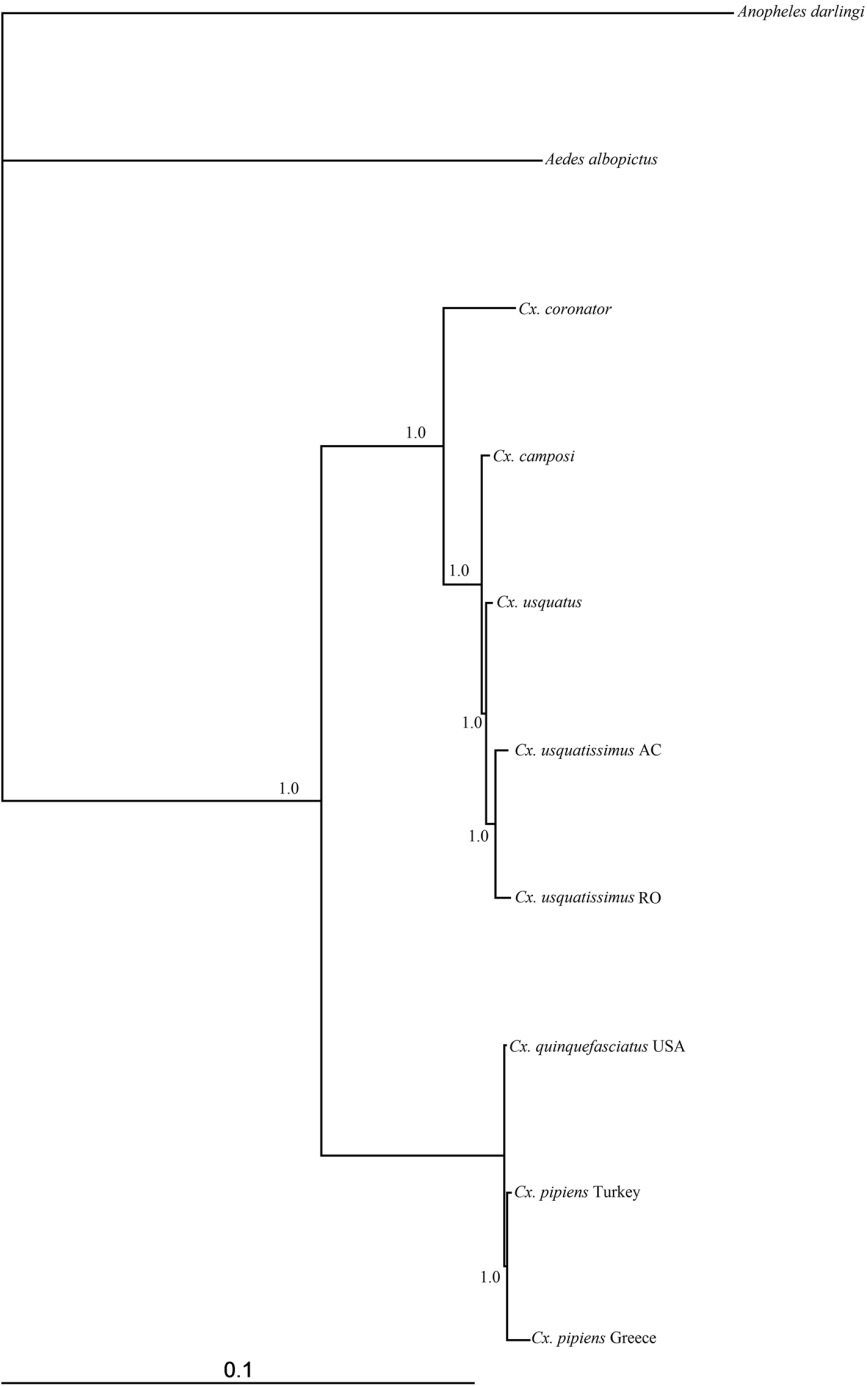
Bayesian topology generated from the analysis using 13 protein-coding genes of four species of the Coronator Group plus *Cx. quinquefasciatus* and *Cx. pipiens*. Numbers placed above the branches indicate the values of Bayesian posterior probabilities. *Aedes albopictus* and *Anopheles darlingi* were employed as outgroups. Complete mitochondrial genomes of *Cx. quinquefasciatus,* two specimens of *Cx. pipens, Ae. albopictus* and *An. darlingi* were downloaded from the GenBank

Best-fit models selected by the Akaike Information Criterion are showed in Additional file 3. The Bayesian topology generated employing the concatenated PCGs (Fig. 4) yielded two strongly supported (100 % posterior probability) monophyletic clades with the Coronator Group as the sister clade of the lineage composed of species of the *Cx. pipiens* complex. Within the clade formed by species of the Coronator Group, the placement of *Cx. coronator* as sister of the lineage leading to *Cx. camposi* plus *Cx. usquatus* plus *Cx. usquatissimus* was strongly supported (100 % Bayesian posterior probability). Additionally, divergence among species from this group under Maximum Composite Likelihood (MCL) was low (Table 5), ranging from 0.2 % between *Cx. usquatus* and *Cx. camposi* to 1.4 % between *Cx. coronator* and *Cx. usquatissimus* (from Acre and Rondônia). The low nucleotide divergence observed among the mitochondrial genes of species of the Coronator Group supports the close phylogenetic relationships observed in previous studies [3–5]. As hypothesized by Lloyd et al. 2012 [55], the high statistical support for the clades suggests that many other species can be included in the PCGs analyses of species of the subgenus *Culex*. However, if more species were included in the study, likely the phylogenetic signal of the mitochondrial genes would be changed. For instance, studies based on morphological phylogeny [56] and *COX1* barcode sequence data of several species of the genus *Culex* [4] were not capable of supporting phylogenetic relationships among species of the Coronator Group and other closely related species such as *Cx. surinamensis* and *Cx. maxi.* Those results suggest that *Cx. surinamensis* and *Cx. maxi* may share their ancestor with current species included in Coronator Group and as such, they should be included in the group. However, further studies need to be developed to address this hypothesis.

**Table 5:** Average of nucleotide divergence under Maximum Composite Likelihood of 13 protein-coding genes of the mitochondrial genome of *Cx. coronator, Cx. usquatus, Cx. camposi,* and *Cx. usquatissimus* and four specimens obtained from GenBank. The averages are expressed as percentages; GenBank accession numbers are indicated between brackets

	1	2	3	4	5	6	7	8
1. *Cx. pipiens* Greece [HQ724615]								
2. *Cx. pipiens* Turkey [HQ724616]	0.3							
3. *Cx. quinquefasciatus* USA [HQ724617]	0.1	0.3						
4. *Cx. usquatissimus* AC	3.9	4.1	3.8					
5. *Cx. usquatissimus* RO	4.0	4.1	3.9	0.3				
6. *Cx. usquatus*	3.9	4.0	3.8	0.3	0.3			
7. *Cx. camposi*	3.8	3.9	3.7	0.3	0.4	0.2		
8. *Cx. coronator*	4.0	4.2	3.9	1.4	1.4	1.3	1.3	

**Fig. 5:**
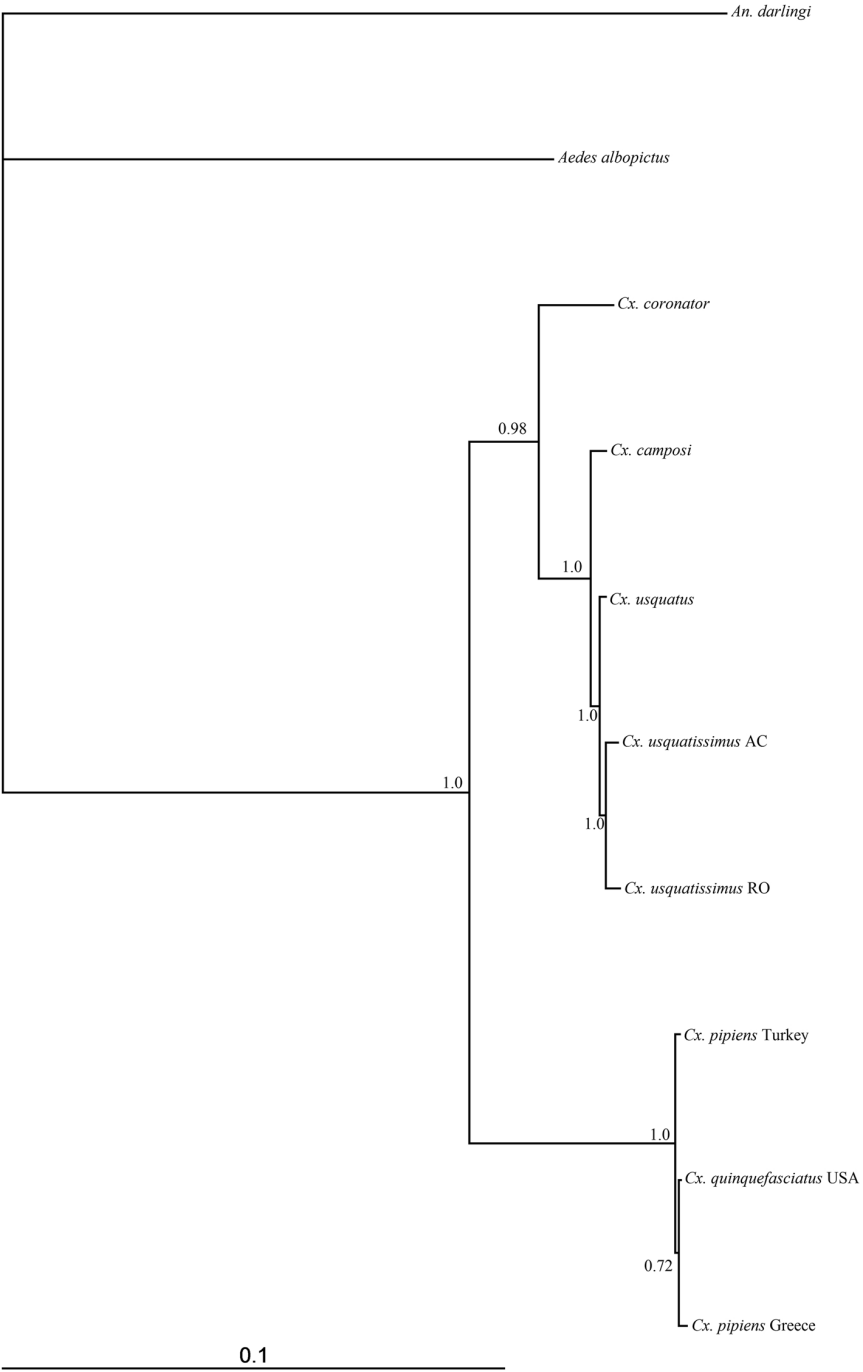
Bayesian topology generated from the analysis using *ATP6, ATP8* and *NADH5* protein-coding genes of four species of the Coronator Group plus two species of *Cx. pipiens* complex. Numbers placed at the branches indicate Bayesian posterior probabilities. *Aedes albopictus* and *Anopheles darlingi* were employed as outgroups. Mitochondrial genomes of *Cx. quinquefasciatus,* two specimens of *Cx. pipens, Ae. albopictus* and *An. darlingi* were downloaded from the GenBank

In order to address the effectiveness of the *ATP6, ATP8* and *NADH5* for reconstructing phylogenetic relationships within the Coronator Group, a Bayesian analysis was performed only with these genes (Fig. 5). Within the Coronator Group, the topology yielded was the same of that performed with all genes, demonstrating the potential of those genes for further evolutionary studies with this group. Interestingly, for *Cx. pipiens* complex species, results of Bayesian analysis recovered a polytomy between *Cx. quinquefasciatus* and *Cx. pipiens.* Those results are important to show how evolutionary behavior of the genes are different among groups of species, even within the same subgenus, and how carefully researchers need to be when selecting a molecular marker.

## Conclusions

The complete mitochondrial genomes of *Cx. usquatus, Cx. usquatissimus*, *Cx. coronator,* and *Cx. camposi* share the same gene order and gene content of other Culicidae species with the mitochondrial genome reported to date. The overall length and the AT content of the four *Culex* species are within the range observed in species of the *Anopheles albitarsis* complex, *Cx. quinquefasciatus,* and *Ae. aegypti.* Except for *trnR* and *trnS1*, the tRNAs were folded into the typical cloverleaf-like structure. Similar to other metazoan and species of the family Culicidae, the control region presented some conservative poly T- stretches, which seems to be involved in the functional roles of mitochondrial genome. Results of the Bayesian phylogeny corroborated the monophyly of the Coronator Group. Nevertheless, new insights on the evolution of the group can be addressed using a larger species sampling, including morphologically similar species and species of other *Culex* subgenera, for example, *Lutzia*, *Phenacomyia* and *Phytothelmatomyia* species. Probably because of the functional roles played in the respiratory chain by the mitochondrial genome, PCGs showed high purifying selection and low nucleotide divergence among the species investigated here.

## Additional files

Additional file 1:
**Mitochondrial genomes organization of species studied.** Format: xls. (XLSX 28 kb) Additional file 2:
**Predicted secondary structures for 20 tRNA genes of Coronator Group.** Colored dots indicate Watson-Crick base pairing; red dots indicate G-C base pairing. Format: png. (PNG 671 kb) Additional file 3:
**Best-fit model chosen for each gene under Akaike information criterion (AIC).** (XLSX 9 kb)
