# Supplementary figures and images for: Mitochondrial genomes and comparative analyses of Culex camposi, Culex coronator, Culex usquatus and Culex usquatissimus (Diptera:Culicidae), members of the coronator group
Source: BMC Genomics. 2015 Oct 21;16:831. doi: 10.1186/s12864-015-1951-0 (PMC4618934; doi:10.1186/s12864-015-1951-0)

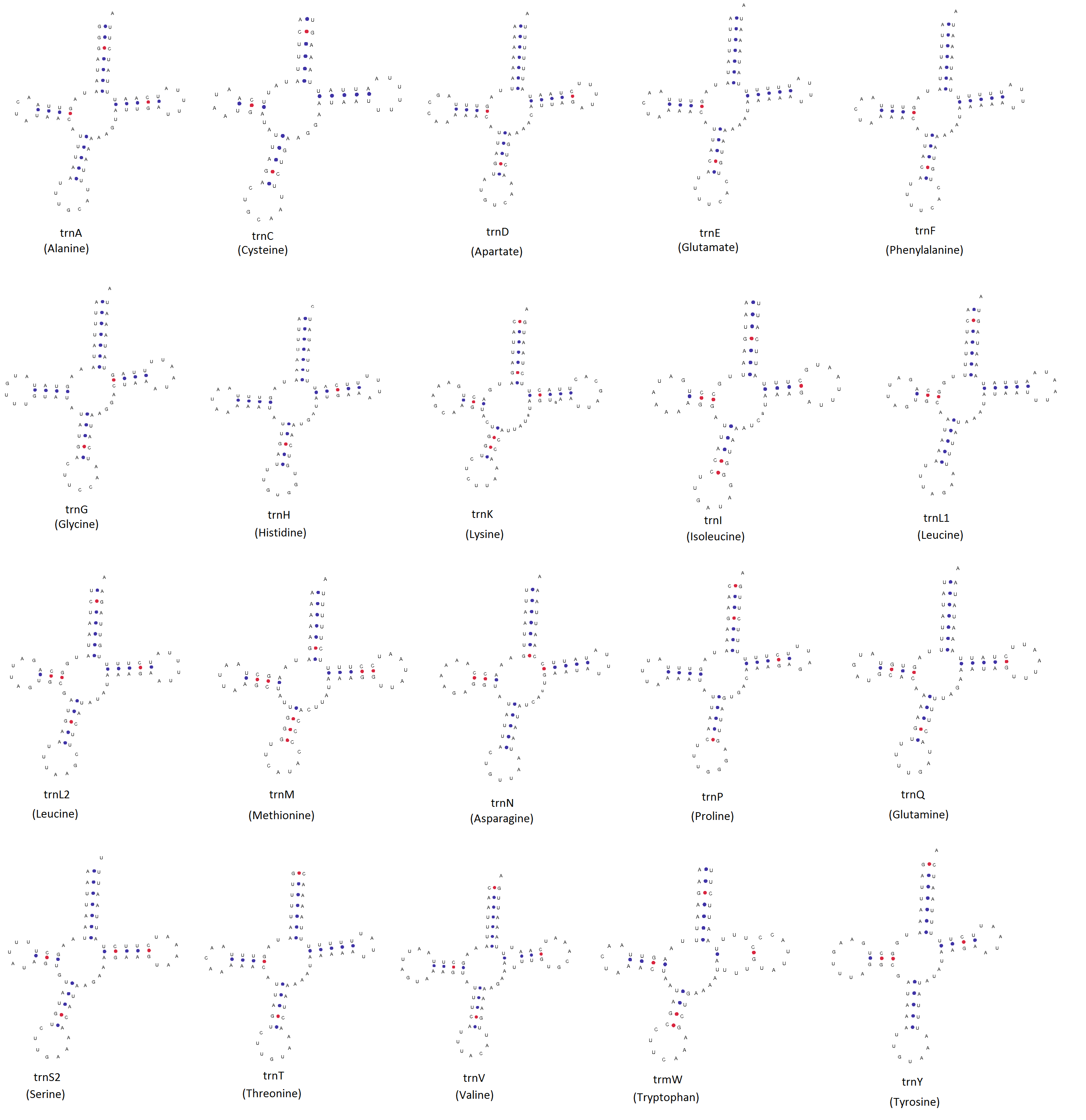

Supplement: Additional file 2: — Predicted secondary structures for 20 tRNA genes of Coronator Group. Colored dots indicate Watson-Crick base pairing; red dots indicate G-C base pairing. Format: png. (PNG 671 kb) [file 12864_2015_1951_MOESM2_ESM.png]
